# β2-Glycoprotein I: structure, mechanisms of autoantibody recognition, and polymorphisms

**DOI:** 10.1016/j.coi.2026.102771

**Published:** 2026-04-07

**Authors:** Suresh Kumar, Nathan Ponzar, Nicola Pozzi

**Affiliations:** Edward A. Doisy Department of Biochemistry and Molecular Biology, Saint Louis University School of Medicine, St. Louis, MO 63104, United States

## Abstract

Identified in the 1990s as the primary target of antiphospholipid antibodies (aPL) in antiphospholipid syndrome (APS), β2-glycoprotein I (β2GPI) remains a central focus in hematology and immunology. Anti-β2GPI antibodies are important not only for diagnosing APS but also play a key role in causing thrombosis and pregnancy complications in these patients. Elucidating the molecular basis of antibody-β2GPI interactions is therefore critical for advancing APS research and has broad implications for understanding related thrombotic autoimmune disorders. In this review, we summarize recent progress on the structural biology of β2GPI, discuss mechanisms of autoantibody recognition, and provide an update on genetic polymorphisms. By resolving longstanding controversies and uncovering new regulatory principles, structural insights are paving the way for targeted approaches aimed at selectively neutralizing pathogenic autoantibodies without broadly impairing coagulation or immune function, offering promising paths toward transformative APS therapies.

## Introduction

Antiphospholipid syndrome (APS) is a systemic autoimmune disorder characterized by recurrent thrombosis and pregnancy morbidity, driven by antiphospholipid (aPL) antibodies [1,2]. Among them, those targeting β2-glycoprotein I (β2GPI) are central to APS pathogenesis [3]. Investigating the structure and function of β2GPI is therefore important for APS researchers and medical professionals caring for these patients.

β2GPI is a well-known 50 kDa plasma glycoprotein that circulates at a concentration of ~0.2 mg/mL. It is highly conserved in mammals and involved in many pathways. Yet despite that, β2GPI deficiency in humans and genetic elimination in mice are compatible with life and produce no overt phenotype [4,5].

In its mature form, β2GPI comprises 326 amino acids organized into five domains (DI-DV) [6]. The first four belong to the complement control protein (CCP) superfamily, each of ~60 residues with two conserved disulfide bonds. In contrast, Domain V (DV) is larger, distinguished by an additional disulfide bond and a 19-residue insertion loop. Four canonical N-glycosylations complete its structure, accounting for ~20% of its mass.

Beyond canonical N-glycosylations, β2GPI undergoes additional post-translational modifications. Carbamylation [7] and various redox-dependent modifications of glycans [8,9], tyrosine [10], and cysteine [11] residues have been reported in recent years. These modifications represent an important area of research because they greatly expand the potential for β2GPI’s functional diversity. However, our understanding of how each of them, individually and in combination, contributes to APS pathogenesis remains limited.

Another biologically important property of β2GPI is its conformational plasticity. Factors such as solution conditions, chemical modifications of its primary structure, and ligand binding could affect β2GPI’s overall shape, thereby regulating its function [12]. Prior observations in analogous CCP-containing proteins support this concept. Factor H, for instance, adopts distinct conformations that are important for its activity as a complement regulator [13,14]. Likewise, complement factor C1 [15,16] and coagulation factor XIII [17] require conformational mobility of CCP domains for activation. In this context, the idea that β2GPI may exist in multiple conformations has captivated investigators and shaped decades of APS research looking to address questions such as: (i) How many conformations are accessible? (ii) Which conformations are functionally relevant? (iii) What molecular events govern transitions between them?

In this article, we review recent advances in the structural biology of β2GPI, discuss their implications for APS pathogenesis, and outline emerging avenues for structure-based therapeutic development.

### The structural landscape of β2-glycoprotein I and the circular model debate: revisiting a long-standing dogma

**Figure 1:**
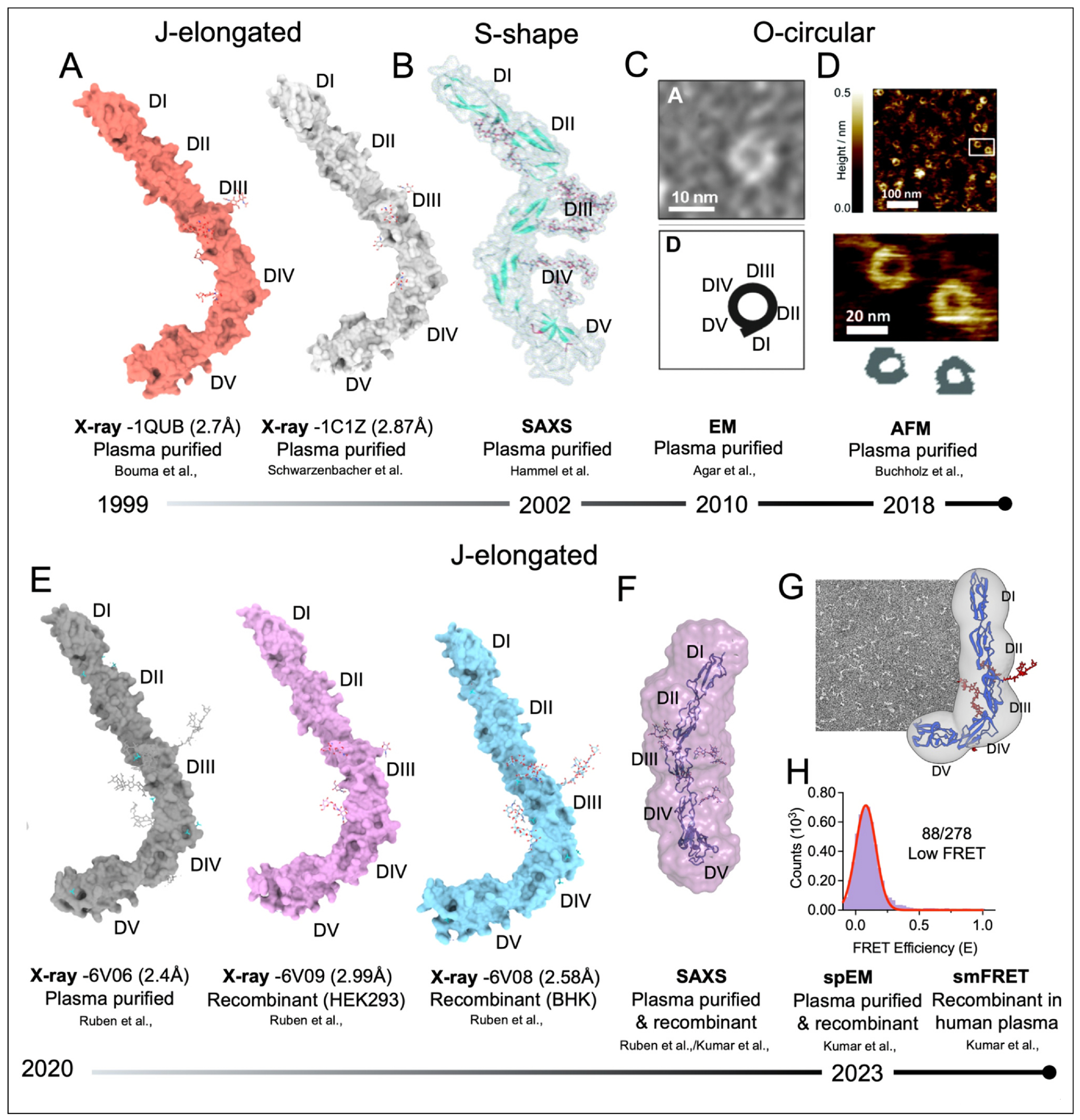
Structural analyses of β2GPI over the years. **(a)** X-ray crystal structures of plasma-purified β2GPI with N-linked glycans are shown as sticks solved in 1999; **(b)** SAXS-derived structure of plasma purified β2GPI proposed in 2002; **(c)** negative stain EM studies in 2010; **(d)** AFM studies in 2018; **(e)** X-ray crystal structures of plasma-purified and recombinant β2GPI solved in 2020, **(f)** SAXS-derived structure of plasma purified and recombinant β2GPI in 2020 (Ruben et al. [24]) and 2021 (Kumar et al. [37]); **(e)** single particle EM, and **(f)** smFRET studies in 2023. Low FRET in smFRET experiment indicates that residues 88 and 278 are located > 90Å apart, consistent with the elongated form model.

The structure of β2GPI was first solved by X-ray crystallography in 1999 by two groups [18,19] (Figure 1a). These studies revealed an elongated architecture with modest bending between DIII and DIV, resulting in a characteristic J-shape. However, questions soon arose about whether this arrangement truly reflects the solution state, given that (i) β2GPI was purified under harsh conditions, and (ii) crystallization occurred under high-salt conditions that are not encountered in plasma.

Potential answers to these questions began to emerge in 2002 when an S-shape form derived from small-angle X-ray scattering (SAXS) was proposed [20] (Figure 1b). However, new and different evidence followed in 2010, when studies using negative stain electron microscopy (EM) (Figure 1c) [21], followed in 2018 by atomic force microscopy (AFM) (Figure 1d) [22], proposed a circular arrangement of the 5 domains in which DI and DV interact to form an O-ring. Discrepancies between these models, especially J-elongated vs O-circular, were attributed to purification/experimental methods, with crystallography studies assumed to favor linearity. Contamination with lipopolysaccharide (LPS) was also proposed at times, since β2GPI bound to LPS appears elongated rather than circular in EM studies [23]. It was therefore concluded that, in plasma, β2GPI is circular and that the J-elongated form likely represents the form bound to membranes, while the S-form may be some kind of intermediate structure.

This view, however, started to shift recently. Beginning in 2020, our group used several traditional and advanced approaches to probe the structural dynamics of β2GPI, including X-ray crystallography [24] (Figure 1e), SAXS [24] (Figure 1f), single-particle electron microscopy (spEM) [25] (Figure 1g), and single-molecule Förster Resonance Energy Transfer (smFRET) [25] (Figure 1h). Extensive investigations using diverse conditions, including those previously proposed to favor circularization, as well as smFRET measurements conducted directly in human plasma [25] — marking the first smFRET study of any protein in human plasma — found no evidence for circular β2GPI species in solution. Instead, these studies consistently show an extended, flexible protein architecture, consistent with monomeric β2GPI primarily adopting a J-elongated form.

Functional studies also support this view [26]. Contrary to predictions of the circular model, but fully consistent with the elongated model, binding of a monoclonal antibody directed against a DI epitope that was thought to be occluded by DV in the circular form was not enhanced by removing DV; rather, it bound to isolated DI with similar affinity, and binding was abolished by targeted mutations within the epitope. This indicates that DI is constitutively exposed and accessible in the solution form of β2GPI. These findings, combined with other considerations recently reviewed by Kumar et al. [27] and the lack of high-resolution structural data, cast considerable doubt on the circular model. It is therefore our opinion that the circular model should no longer be used to describe the solution structure of monomeric β2GPI as inconsistent with structural and functional evidence.

**Figure 2:**
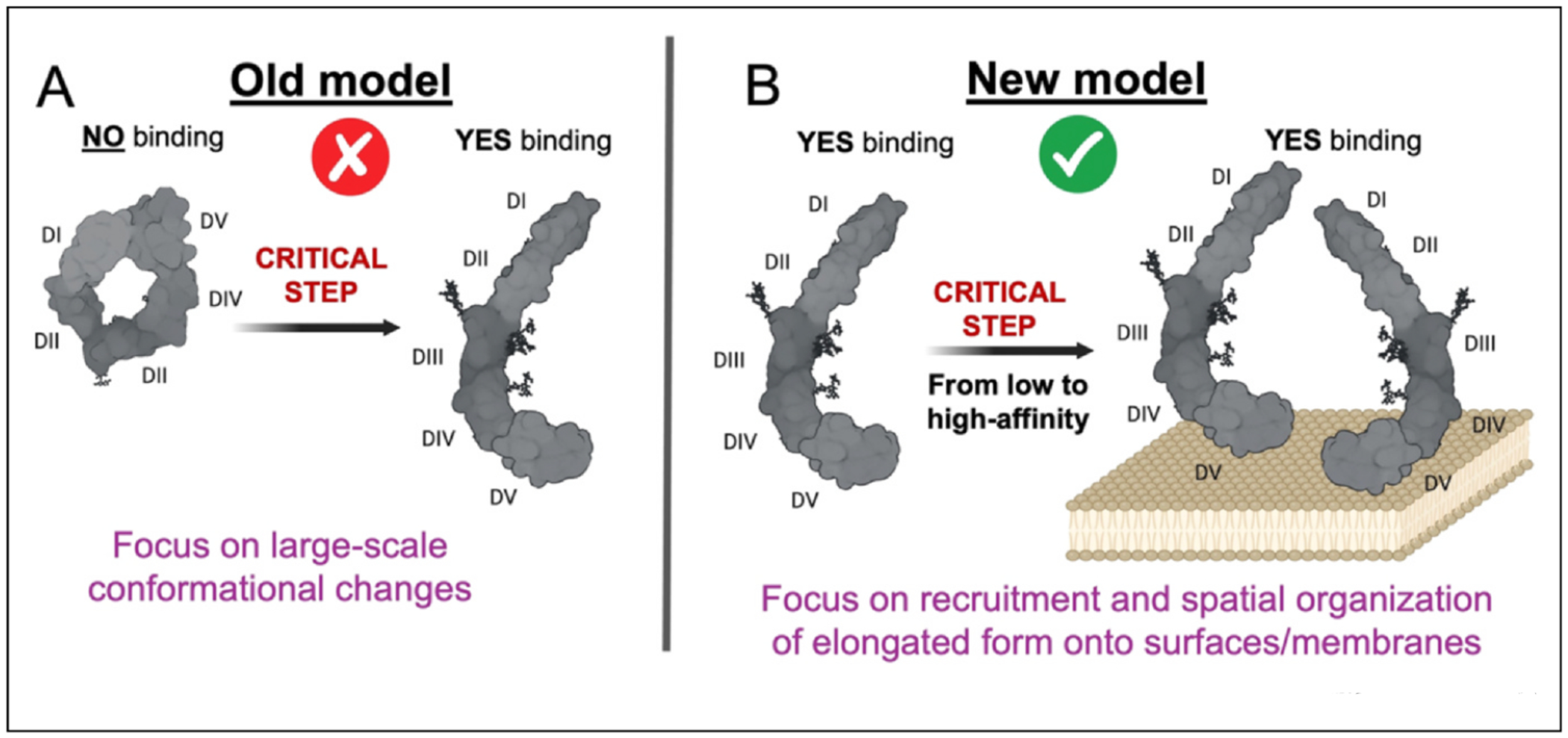
Autoantibody binding: old model vs new model. **(a)** *Old model*: the critical step for aPL antibody recognition is a large-scale conformational transition from a circular to an elongated form. **(b)** *New model*: the critical step is not a large conformational shift but rather the transition from low-affinity to high-affinity complexes driven by DV and avidity, as described in this review.

Embracing this change is important for two reasons. First, it allows for a more balanced and careful interpretation of prior literature, particularly studies in which β2GPI was thought to be circular, but which we now know is not the case. Second, the fact that soluble β2GPI is elongated and autoantibodies can bind to it significantly changes our perspective on the early, critical events that drive disease and advances new ideas on how to neutralize pathogenic autoantibodies. It is no longer about large-scale conformational changes (Figure 2a) but rather how the elongated form becomes pathogenic (Figure 2b).

While introducing a paradigm shift, it is important to keep in mind that the elongated model is not static and does not exclude alternative conformational arrangements or forms of β2GPI. Local structural rearrangements may fine-tune autoantibody binding, and rapid domain movements within the elongated structure, such as those monitored by smFRET, may evetually give rise to the transient appearance of various S-shaped forms observed in theoretical studies [28]. However, the existence of such computer-generated structures and their functional significance, if any, remains a matter of debate and should be proven in a test tube using rigorous, orthogonal structural approaches.

Even if the elongated monomer represents the predominant soluble form, understanding the origin of the previously reported circular conformation remains relevant. Several approaches could help clarify this issue. Sharing protein preparations among different groups, including those used in the original studies, may provide valuable insights. Since EM and AFM require surface-immobilized β2GPI for imaging, investigating the influence of specific surfaces could also be informative. Exploring alternative plasma-purification workflows may likewise shed light on this issue. Although plasma-purified and recombinant β2GPI from HEK293 and Baby Hamster Kidney cells are essentially always monomeric in our hands, early studies suggest β2GPI may form multimers. While in some cases multimers were explicitly induced by oxidative treatment [29], in other cases they were not [30,31]. Finally, the possibility that β2GPI associates with other plasma proteins warrants consideration. For example, prior work reported interactions between β2GPI and von Willebrand factor [32], FXI/XIa [33], thrombin [34], and, more recently, preliminary work by Acquasaliente et al. suggests that β2GPI can also bind to fibrinogen [35]. Thus, while monomeric β2GPI is elongated, the existence of alternative forms, multimers, and complexes warrants renewed investigation to fully understand its role in human physiology and APS.

### Affinity, avidity, domain V, and the ‘zip code’ model

A key factor in understanding why the circular model was so well received by the community and persisted for more than a decade lies in a seminal observation: although immune complexes readily form when β2GPI associates with phospholipids or other suitable surfaces, they are rarely detected in patients’ plasma. A β2GPI molecule that is incapable of recognizing aPL in solution but switches to an active conformation when bound to surfaces offered a conceptually elegant explanation. However, this explanation is not the only plausible one.

Two aspects of antibody biology are particularly important in APS: autoantibody affinity and autoantibody titers. Unlike antibodies that have undergone affinity maturation and bind their antigens with nanomolar affinity, aPL antibodies generally exhibit lower affinities, in the micromolar range [36]. This feature stems from rapid association-dissociation kinetics and electrostatically driven complex assembly [24,26,37]. At the same time, aPL antibody titers in plasma typically represent ~0.5–1% of total IgG, corresponding to submicromolar concentrations. The combination of micromolar affinity and sub-micromolar antibody levels explains why soluble immune complexes are rarely detected. It is not that the complexes cannot form. They can form, but they are short-lived as they easily fall apart.

Low affinity and low antibody titers address one part of the problem, specifically why immune complexes are rarely detected in plasma. However, they do not explain how they form at pathogenic sites. The answer to that question comes from the interplay between avidity and DV. DV contains an insertion loop that confers distinctive properties, most notably its ability to bind anionic surfaces, particularly phospholipids. This interaction sharply increases local antigen density and orients the remaining β2GPI domains outward from the membrane. Under these conditions, low-affinity antibodies gain functional avidity by several orders of magnitude: association becomes more favorable while dissociation is less likely [38]. Thus, a low-affinity aPL antibody effectively behaves as a high-affinity antibody provided that 1) DV anchors β2GPI to phospholipids/surfaces and 2) two β2GPI molecules are sufficiently close for the antibody to bind them simultaneously. Under these circumstances, large-scale conformational rearrangements of domains in β2GPI are not required to explain enhanced aPL binding to surface-bound antigens. Instead, DV becomes key. In a way, DV acts as a molecular ‘zip code’ in that it directs β2GPI to specific cellular and vascular microenvironments and controls its spatial arrangement, thus allowing aPL antibodies to engage it effectively.

Perhaps one of the most compelling examples supporting this model is the behavior of Bavituximab, a chimeric monoclonal antibody under evaluation as an anticancer agent [39]. Bavituximab binds soluble β2GPI weakly but specifically accumulates in tumor lesions because these sites expose high levels of phosphatidylserine, which recruit β2GPI [40]. A similar concept, but with a different therapeutic goal, was recently disclosed by Macor et al. [41]. The authors coated fibrinolytic nanobubbles with a monoclonal antibody that weakly recognizes β2GPI in solution but binds to it strongly when bound to surfaces, including vascular endothelial cells. Harnessing local β2GPI enrichment enabled targeted fibrinolysis at thrombotic sites. These examples highlight not only the relevance of the ‘zip code’ model but also the emerging therapeutic potential of exploiting β2GPI’s localization properties.

Importantly, although inspired by β2GPI biology, the ‘zip code’ model reflects fundamental principles of antibody recognition and should extend to other aPL antibodies and to low-affinity antibodies more broadly. For instance, we recently showed that POmAb, a type I anti-prothrombin antibody, binds the open form of mouse prothrombin with a low affinity, allowing selective targeting of endothelial-bound rather than soluble open prothrombin. This confers anticoagulant activity without increasing bleeding risk [42]. Similarly, next-generation bispecific antibodies for hemophilia rely on the low intrinsic affinity of each arm for their soluble targets but achieve high functional avidity when the antigens are appropriately oriented on a surface to correct the bleeding defect [43,44].

In conclusion, the binding of aPL antibodies to their targets follows fundamental biophysical principles of affinity and avidity, and requires precise spatial organization of antigens on a suitable surface. These principles apply broadly to antibody biology but are also applicable to other biological systems designed to act locally rather than systemically such as the coagulation and complement cascades. A final, often overlooked implication for therapeutic strategy is that higher affinity is not universally beneficial; in some physiological settings, moderate affinity allows more controlled and context-appropriate engagement. In this sense, when considering affinity, less can indeed be more.

### Structural insights into DV-interactions, redox regulation, and proteolysis

The critical role of DV highlighted above justifies continued investigation into its structure and interactome.

**Figure 3:**
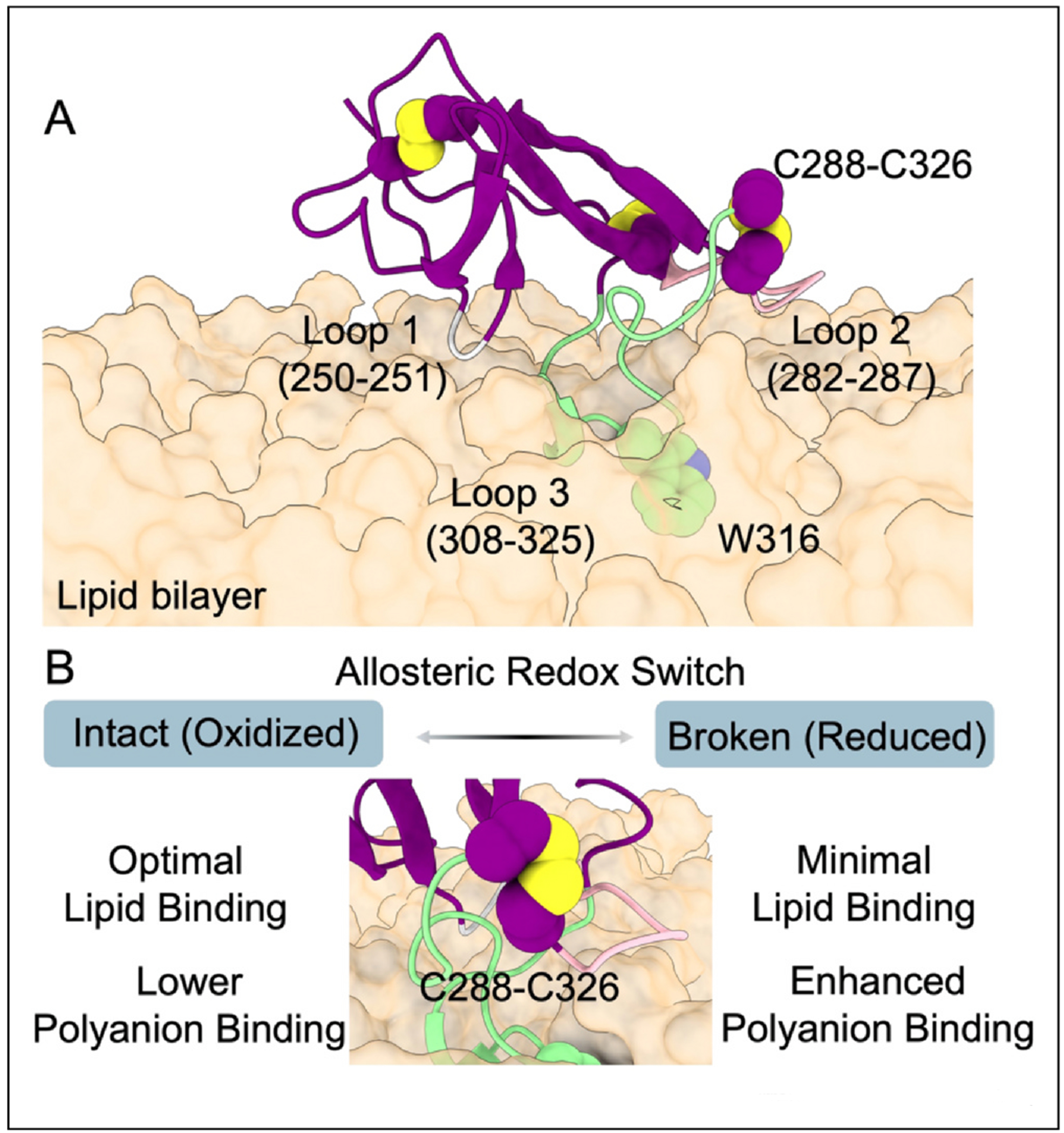
Structural basis of DV function and regulation. **(a)** DV bound to an anionic membrane in its most stable configuration, with loop 3 inserting into the bilayer and positioning W316 within the membrane, as described by Hasdemir et al. The other two loops, corresponding residues, and the C-terminal disulfide bond (C288-C326) are highlighted. **(b)** Functional role of the C288-C326 disulfide bond and the consequences of its allosteric regulation.

Anionic phospholipids, such as phosphatidylserine and cardiolipin, are major components of cell membranes and key ligands for DV. Recent studies have begun to resolve the interaction of DV with phosphatidylserine- containing membranes at atomic resolution (Figure 3a), defining how DV approaches and interacts with these surfaces [45]. These analyses have also revealed a novel binding contribution from lysine pair 250/251 and identified an additional membrane-bound orientation. For now, these analyses remain limited to model membranes with simplified chemical compositions. However, growing knowledge of membrane composition in different cell types in APS patients [46], identification of new coagulation bioactive phospholipids [47], and advances in computing power [48] will inform future theoretical and experimental work aimed at examining how membrane composition influences β2GPI recruitment to specific cells and vascular niches, which is a critical and unresolved question in the field.

Another key insight from recent structural work is the potential for redox regulation (Figure 3a). Building upon the seminal discovery that β2GPI is a substrate of the protein disulfide isomerase (PDI) [49], we found that the C288-C326 disulfide bond acts as an allosteric redox switch, such that its reduction negatively impacts phospholipid binding and, consequently, aPL recognition [37]. This is because stable binding of DV to membranes is mediated by the insertion of the loop 308–325, which itself is stabilized by the C-terminal C288-C326 disulfide bond [45].

Beyond anionic phospholipids, β2GPI associates with various polyanions, including heparin, DNA, neutrophil extracellular traps (NETs), and polyphosphates *(our unpublished data)*. Interestingly, while the absence of the C288-C326 bond reduces phospholipid binding, it increases its affinity for heparin [37]. This disulfide bond, therefore, functions as a molecular switch regulating β2GPI’s adhesive preferences across distinct surfaces (Figure 3b). In this context, recently published mechanistic studies on the interaction of β2GPI with NETs and Platelet Factor 4 (PF4) are of great interest [50,51]. β2GPI bound to NETs via PF4 might recruit pathogenic aPL antibodies to the NET surface, thereby creating a vicious cycle that intensifies prothrombotic and proinflammatory responses.

Regulation of DV function is not limited to redox modifications but also involves proteolytic cleavage. Early studies demonstrated that *in vivo* cleavage of β2GPI at K317-T318 by plasmin produces a ‘clipped’ protein that retains an intact C288–C326 disulfide bond [52,53]. This form has altered phospholipid-binding properties and functions [52,53]. However, if the same cleavage occurs in the reduced form of β2GPI, the resulting product will be shortened by eight residues (T318-C326). Although recent studies have begun to explore structural and functional differences between these proteolytic forms [28], insufficient characterization of β2GPI species complicates the interpretation of these results and warrants further investigation.

The interplay between DV redox states and proteolytic degradation is especially compelling from an immunology perspective. Intact β2GPI, although functioning as an LPS scavenger, does not exhibit direct antibacterial properties. In contrast, positively charged peptides generated from DV following proteolytic cleavage display broad-spectrum antibacterial activity [54], likely forming pores that disrupt bacteria’s membranes. Given that the C288-C326 disulfide bond may or may not be present, the generation and release of these peptides likely vary across redox and proteolytic conditions. Thus, redox regulation of β2GPI may be linked to immunity and, perhaps, autoimmunity as well. Interestingly, autoantibodies against DV are transiently detected during infection [55], and anti-DV antibodies from APS patients do not appear to be pathogenic when infused into animals [56]. Consistent with this, recent studies have shown that detection of anti-DV antibodies might help identify APS patients at lower risk than those with antibodies reacting against other domains [57], such as those targeting specific epitopes in DI [26]. One theory is that DV-derived peptides are initially beneficial during infection, but, in genetically or immunologically predisposed individuals, prolonged or excessive exposure could sensitize the immune system and contribute to persistent aPL generation. Although this model remains to be tested, a recent study showed that aPL responses triggered by infection are mediated by tissue factor [58], providing a new mechanistic link between infections and aPL generation and suggesting actionable therapeutic opportunities. Furthermore, because tissue factor activity, like β2GPI, is regulated by PDI, targeting PDI may represent a promising therapeutic strategy for APS [59].

Another key aspect of DV biology is its proposed role in receptor binding. Several candidates have been suggested, including ApoER2, Toll-like receptors 2 and 4, glycoprotein Ibα, and annexin A2. While some biochemical evidence supports these interactions, structural and mechanistic validation is lacking. The only structural evidence dates back 15 years and reports interactions between four ligand-binding LA modules of LDLR and ApoER2 and DV [60]. Importantly, this study indicated that LA modules and phospholipid association are mutually exclusive binders, raising unresolved questions about whether membrane engagement precedes receptor binding. Furthermore, receptor clustering and dimerization are recognized as critical for aPL antibody function, yet their dynamics remain poorly understood. Future biophysical, cryo-EM, and cryo-ET investigations will be essential to elucidate the molecular basis of β2GPI-receptor interactions, providing fundamental insights into recognition mechanisms and their functional roles in APS pathogenesis.

### Polymorphisms and their potential functional impact

**Figure 4:**
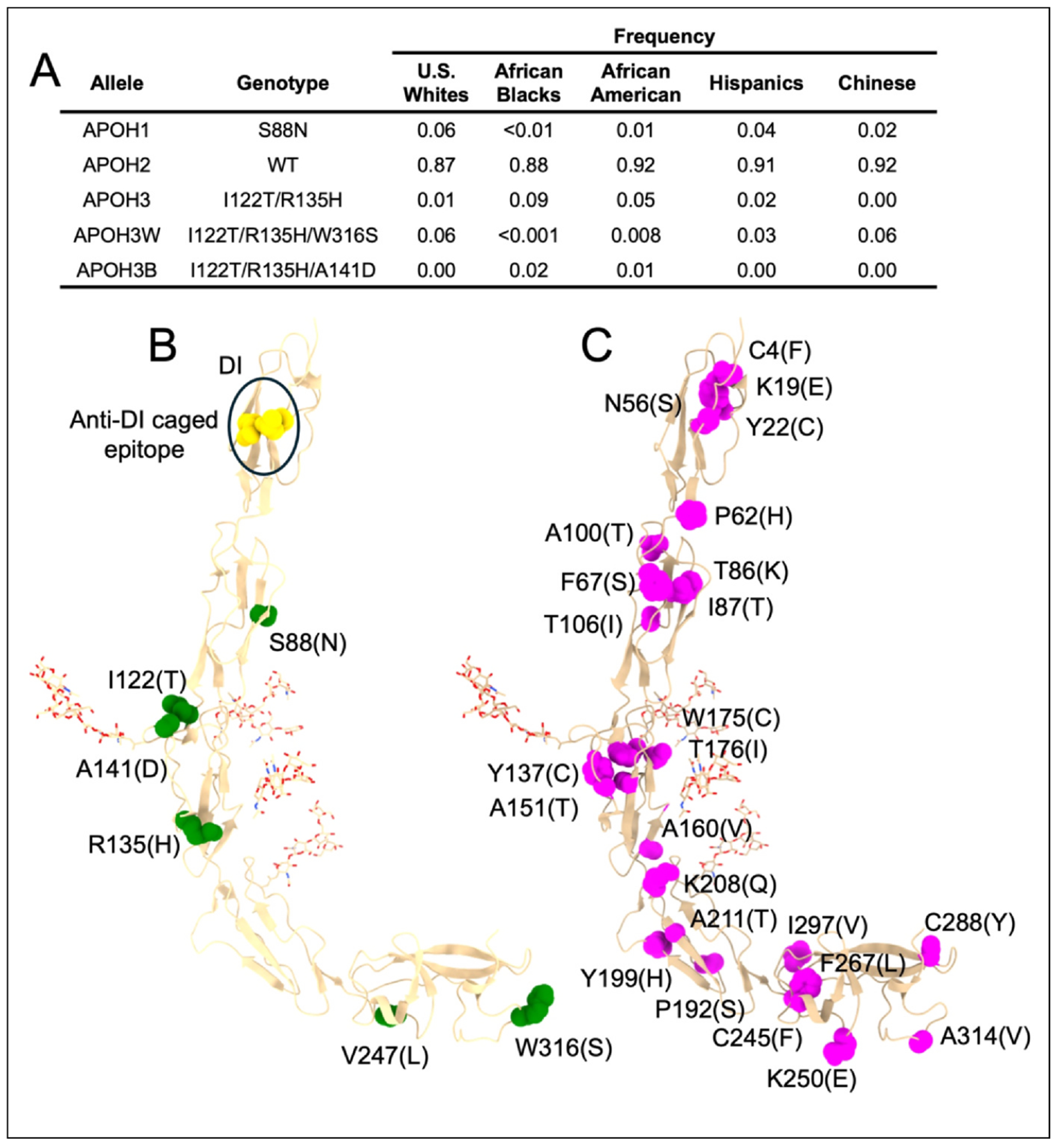
β2GPI polymorphisms. **(a)** Summary of APOH allele frequencies across major racial groups. **(b)** Structural mapping of APOH allele frequencies and the common polymorphism V247L (green spheres) on the crystal structure of plasma β2GPI (PDB: 6V06). Residues R43, N56, and T57 in DI forming the cage structure are shown in yellow. Note that residue 88 is located > 35 Å from the cage and oriented toward the right side of the molecule, whereas the cage lies on the left. **(c)** Mapping of missense APOH polymorphisms reported in ClinVar. In both panels B and C, amino acid substitutions are indicated in parentheses.

β2GPI is encoded by the APOH gene on chromosome 17. In the late 1980s through the early 2000s [61,62], five major allelic variants of this gene were identified. These variants, called APOH1, APOH2, APOH3, APOH3W, and APOH3B, differ by discrete amino acid substitutions and exhibit distinct population frequencies (Figure 4a).

APOH2 encodes the most prevalent isoform (~87%) and is considered the wild type. APOH1 carries a single substitution, S88N, whereas APOH3 harbors two mutations, I122T and R135H. APOH3W and APOH3B are closely related to APOH3 but differ in two amino acid substitutions: W316S for APOH3W and A141D for APOH3B. The allele APOH3B is enriched in individuals of African descent, whereas APOH3W is enriched in Caucasian populations.

The S88N substitution in APOH2 introduces a new glycosylation site, confirmed by mass spectrometry [63]. Located in DII, this modification may influence the recognition of nearby epitopes by autoantibodies. However, at least for most anti-DI antibodies found in thrombotic APS patients [26], this seems unlikely to occur without major rearrangements of the protein structure, given that such antibodies target a conformational epitope located far away from this site (Figure 4b).

In contrast to APOH2, mutations in APOH3 occur within DIII. DIII is extensively glycosylated in the wild-type protein, and this feature likely limits autoantibody reactivity to it. In this context, the A141D substitution in APOH3B is interesting as it lies near the major glycosylation site at N143, raising the possibility that it disrupts normal post-translational modifications and exposes neo-epitopes. Also interesting is that APOH3B shows the greatest sequence similarity to the chimpanzee ortholog, and these animals exhibit higher baseline aPL positivity (up to 54%) than humans (10–11%) [64]. It would be interesting to see if some of them target DIII.

The W316S mutation in APOH3W resides in DV. Prior studies have indicated that it impairs phospholipid binding [65], making it of considerable interest in the context of APS pathogenesis. In fact, according to the ‘zip code’ model, individuals carrying this mutation may be protected from anti-β2GPI-driven pathogenic effects. A human study explored this hypothesis [66], but conclusions remain limited by small sample size. Nonetheless, since no abnormalities were reported among homozygous carriers, it was concluded that this mutation is compatible with a healthy life.

In addition to these five allelic variants, another well-recognized polymorphism is V247L [62]. Although occurring across multiple alleles, it is most often associated with APOH2 and APOH3B. This substitution lies within DV, but in a region not predicted to interact with membranes. Biochemical analyses suggest local structural differences between wild-type and V247L [67,68] that may influence local dynamics or receptor interactions. However, these findings, as well as the association between V247L and enhanced aPL antibody generation, require further confirmation.

Beyond well-known allelic variants and polymorphisms, the polymorphic landscape of β2GPI is expected to continue to expand over time thanks to advances in sequencing technology. A search in the ClinVar database as of November 2025 show 28 additional missense variants (https://www.ncbi.nlm.nih.gov/clinvar) most of whom, except for one (K19E) [69], remain of uncertain significance.

Structural mapping of the 28 variants reveals a scattered distribution across the five domains, with minor local clustering in DI, DII, and DIII (Figure 4c). Of those, N56S, K250E, C288Y, and A314V stand out based on recent structural insights. N56 in DI plays a key role in the ‘cage’ recognized by certain anti-DI antibodies [26]. The N56S substitution may reduce recognition by these antibodies, potentially offering protection; however, structural disruption of DI could conversely promote novel aPL specificities. Residue K250 in DV has recently been implicated in phosphatidylserine binding [45]. Therefore, K250E is expected to weaken membrane interactions due to charge reversal. The mutation C288Y in DV eliminates one of the two cysteines necessary for the C288-C326 allosteric disulfide bond [37], likely enforcing a constitutively ‘reduced-like’ β2GPI conformation and resulting in loss of phospholipid binding. Finally, A314V, located within the phospholipid insertion loop in DV, may enhance phospholipid binding and, thus, unlike C288Y, could represent a gain-of- function variant.

In future studies, testing the role of these polymorphisms will be important to close the gap between genetic and functional aspects and to gain insight into how chemical diversity modulates β2GPI function and autoantibody recognition. Although current evidence does not support routine APOH genotyping in clinical practice, declining sequencing costs and expanding biobank resources may soon make such analyses feasible. In turn, this could enable more refined APS phenotyping, better risk stratification, and the development of personalized therapeutic strategies.

## Conclusions

Β_2_GPI remains a central player in APS. Recent advances in structural biology have illuminated key features of this protein and, through the ‘zip code’ model, provide a robust framework for future investigations and development of targeted therapeutics. Nevertheless, critical questions persist, underscoring the need for continued research. Given the complexity of APS, meaningful progress will hinge on collaborative studies that integrate an expanding arsenal of modern structural, cellular, and omics approaches. Success in this endeavor could usher in a new era for APS and related autoimmune disorders, one in which medicines are designed and developed using structure-based principles.
